# Mode of death in elderly and super‐elderly patients with acute heart failure: Insights from Japanese heart failure registry

**DOI:** 10.1002/clc.23619

**Published:** 2021-05-08

**Authors:** Kensuke Takabayashi, Shouji Kitaguchi, Takashi Yamamoto, Kotoe Takenaka, Hiroyuki Takenaka, Ryoko Fujita, Miyuki Okuda, Osamu Nakajima, Hitoshi Koito, Yuka Terasaki, Tetsuhisa Kitamura, Ryuji Nohara

**Affiliations:** ^1^ Department of Cardiology Hirakata Kohsai Hospital Osaka Japan; ^2^ Department of Internal medicine Osaka Hospital Osaka Japan; ^3^ Department of Cardiology Hirakata City Hospital Osaka Japan; ^4^ Department of Cardiology Otokoyama Hospital Kyoto Japan; ^5^ Department of Internal medicine Arisawa General Hospital Osaka Japan; ^6^ Department of Social and Environmental Medicine, Graduate School of Medicine Osaka University Osaka Japan

**Keywords:** cause of death, heart failure death, infection death, mortality, older patients

## Abstract

**Background:**

In Japan, both the prevalence of the elderly and super‐elderly and those of acute heart failure (AHF) have been increasing rapidly.

**Methods:**

This registry was a prospective multicenter cohort, which enrolled a total of 1253 patients with AHF. In this study, 1117 patients' follow‐up data were available and were categorized into three groups according to age: <75 years old (nonelderly), 75–84 years old (elderly), and ≥ 85 years old (super‐elderly). The endpoint was defined as all‐cause death and each mode of death after discharge during the 3‐years follow‐up period.

**Results:**

Based on the Kaplan–Meier analysis, a gradually increased risk of all‐cause death according to age was found. Among the three groups, the proportion of HF death was of similar trend; however, the proportion of infection death was higher in elderly and super‐elderly patients. After adjusting for potentially confounding effects using the Cox and Fine–Gray model, the hazard ratio (HR) of all‐cause death increased significantly in elderly and super‐elderly patients (HR, 2.60; 95% confidence interval [CI], 1.93–3.54 and HR, 5.04; 95% CI, 3.72–6.92, respectively), when compared with nonelderly patients. The highest sub‐distribution HR in detailed mode of death was infection death in elderly and super‐elderly patients (HR, 4.25; 95% CI, 1.75–10.33 and HR, 10.10; 95% CI, 3.78–27.03, respectively).

**Conclusions:**

In this population, the risk of all‐cause death was found to increase in elderly and super‐elderly. Elderly patients and especially super‐elderly patients with AHF were at a higher risk for noncardiovascular death, especially infection death.

## 1 INTRODUCTION

In 2018, life expectancy in Japan was 87.3 and 81.2 years old in females and males, respectively.^1^ Japan has the highest proportion of elderly and super‐elderly people in the world. Both the prevalence of the elderly and super‐elderly and those of heart failure (HF) have been increasing rapidly in most countries worldwide.^2^, ^3^ Improvements in the treatment of HF are shown in many guidelines, and patients with HF have better outcomes.^4^, ^5^ However, many patients with acute HF (AHF) were older and had many comorbidities, and previous large randomized clinical trials had a limitation of a direct adoption for all patients in real‐world settings because of various exclusion criteria.^6^, ^7^ Therefore, information for detailed prognosis in elderly and super‐elderly patients are limited.

Although well‐established medical and support therapies improved survival discharge and prolonged prognosis in patients with AHF, morbidity and mortality are still high especially in elderly patients with AHF.^8^, ^9^, ^10^ Some single‐center studies showed detailed mode of death in elderly and super‐elderly patients with AHF.^11^, ^12^ Elderly and super‐elderly patients with AHF had the tendency to have noncardiovascular death and infection death. However, few multicenter prospective studies describing the detailed mode of death during long‐term follow‐up period still exist. Further data and understanding about the detailed mode of death in patients with AHF are necessary.

The Kitakawachi Clinical Background and Outcome of Heart Failure (KICKOFF) Registry was a prospective, multicenter registry of Japanese patients with AHF.^13^ A total of 13 hospitals in Osaka, Japan, participated in the registry. This study aimed to use the database to evaluate the detailed mode of death in patients with AHF according to age especially focusing on elderly and super‐elderly patients.

## 2 METHODS

### 2.1 Study design

The AHF patients' data from the KICKOFF Registry, which were registered between April 2015 and August 2017, were analyzed. A total of 13 hospitals, consisting of one cardiovascular center and 12 small‐ or medium‐sized hospitals in the north of Kitakawachi (Hirakata City, Neyagawa City, and Katano City) and Yawata, participated in the registry.^13^ The patients were diagnosed with HF based on the Framingham criteria when the presence of at least two of the major criteria, or one major and two minor criteria, in each hospitalization was identified.^14^ This registry has no exclusion criteria. The KICKOFF Registry is registered with the University Hospital Medical Information Network Clinical Trials Registry in Japan (UMIN000016850). The registry design has been described in detail elsewhere.^7^, ^13^ The study protocol was in accordance with the ethical guidelines of the 1975 Declaration of Helsinki and was approved by the ethics committee of the Hirakata Kohsai Hospital (Osaka, Japan). All participants provided written informed consent before their enrollment in the study. The study did not affect any treatment or any other methods of outpatient care.

### 2.2 Patient and outcome data definitions

Among the 1253 patients enrolled in this study, 1118 were alive at discharge, and one patient was excluded because of missed follow‐up data. Finally, 1117 patients were available and categorized into three groups according to age: <75 years old (nonelderly), 75–84 years old (elderly), and ≥ 85 years old (super‐elderly) with reference to previous studies.^3^, ^11^, ^12^ The other definitions of each comorbidity were described in our previous paper.^13^

Follow‐up data were primarily collected by reviewing hospital records, and additional follow‐up information was obtained via telephone or mail contact with the patients or their relatives collected at 6 months, 1 year, 2 years, and 3 years after discharge. All‐cause death and detailed mode of death during the follow‐up period were evaluated as adverse outcome. All‐cause death was divided into two groups: cardiovascular death and noncardiovascular death. Cardiovascular death was defined as death due to HF, sudden death, acute coronary syndrome (ACS), stroke or intracranial hemorrhage, and other cardiac death causes. HF death was defined as a death that occurred as a result of worsening or intractable HF. Sudden death was defined as an unexplained death of a previously stable patient. ACS included acute myocardial infarction and unstable angina. Noncardiovascular death was defined as death due to infection, cancer, renal or liver failure, and other causes. The mode of death in each patient was defined as the critical condition that initiated the train of events leading directly to death. Only one underlying mode of death was applied to each death event. The mode of death was classified as unknown when the mode of death could not be classified, or the detailed information of death was lacking.

### 2.3 Statistical analysis

The clinical baseline characteristics were compared among the three groups using Cochran–Armitage tests and Dunnett's tests, for categorical variables and continuous variables, respectively. Continuous variables are expressed as a mean ± *SD* or interquartile range, and categorical variables are expressed as numbers and percentages. Crude mode of death rates per 100 person‐years with 95% confidence interval (CI) was calculated in each group. The Kaplan–Meier method was applied to evaluate the cumulative incidences of all‐cause death, cardiovascular death, noncardiovascular death, HF death, and infection death. The differences were evaluated by conducting a log‐rank test and the Bonferroni method for multiple comparisons.

Moreover, multivariate analysis was conducted using a Cox proportional hazard model to evaluate the association among the three groups and the incidence of the all‐cause death. Hazard ratio (HR) with 95% CI was also calculated. Potentially confounding effects were also adjusted in multivariable models that were considered to be associated with the clinical outcomes, including sex and comorbidities (yes/no): history of HF, coronary artery disease (CAD), valvular disease, cardiomyopathy, hypertension, diabetes mellitus, atrial fibrillation, chronic kidney disease (CKD), and stroke. Furthermore, to evaluate the effect of clinical factors, the covariates were selected as follows: sex, eGFR, BNP and prescriptions at discharge (yes/no): renin‐angiotensin system (RAS) inhibitors, β‐blocker, diuretic, mineralocorticoid receptor antagonist (MRA), calcium‐channel blocker, oral inotropic agent, digitalis and oral diabetic agent. Included variables were assumed to be time independent from the discharge.^10^, ^15^

Competing risk analysis was conducted to obtain a more insightful interpretation of the impact of the detailed mode of death. First, two types of death, cardiovascular death and noncardiovascular death, were treated as competing events. Second, more details of the four types of death (HF death, cardiovascular death without HF death, infection death, and noncardiovascular death without infection death) were also treated as competing events. Confounder‐adjusted analysis was applied using the Fine–Gray model,^16^ which is a Cox‐type regression analysis for competing risk analysis. Sub‐distribution HRs with 95% CI were estimated. Variables adjusted in this model were the same as those in the Cox proportional hazard model.

All statistical analyses were conducted by JMP version 14 (SAS Institute, Cary, NC, USA) and EZR on R commander Version 1.41 in this study.^17^ p < .05 was considered statistically significant.

## 3 RESULTS

Among the 1117 patients, 393 (35.2%), 401 (35.9%), and 323 (28.9%) were nonelderly, elderly, and super‐elderly patients with AHF, respectively. All follow‐up data were available as of August 2020. The median follow‐up period was 1078 days (interquartile range, 430–1160 days).

Table 1 shows the baseline clinical characteristics. Overall, 51.1% of the patients were males, and the mean age was 77.3 years. The proportion of males tended to be lower with increasing age groups. No significant difference was observed in the proportion of previously diagnosed HF. Super‐elderly patients had the lowest proportion of CAD, cardiomyopathy, and diabetes mellitus. By contrast, super‐elderly patients had the highest proportion of valvular disease, CKD, and stroke. With increasing age groups, LVEF tended to be higher, whereas BMI and eGFR tended to be lower.

**TABLE 1:** Baseline characteristics of the study population as stratified according to age

	All population	Nonelderly	Elderly	Super‐elderly	p trend
*N*	1117	393	401	323	
Age (years)	77.3 ± 5.7	65.0 ± 8.6	79.7 ± 2.9	89.5 ± 3.6	<.001
Male	571 (51.1%)	259 (65.9%)	211 (52.6%)	101 (31.3%)	<.001
BMI (kg/m^2^)	21.9 ± 4.0	23.2 ± 4.6	21.7 ± 3.5	20.5 ± 3.6	<.001
Comorbidities					
Hear failure	654 (58.6%)	229 (58.3%)	235 (58.6%)	190 (58.8%)	0.880
Coronary artery disease	319 (28.6%)	117 (29.8%)	129 (32.2%)	73 (22.6%)	.046
Valvular disease	121 (30.2%)	96 (24.4%)	121 (30.2%)	123 (38.1%)	<.001
Cardiomyopathy	167 (15.0%)	85 (21.6%)	56 (14.0%)	26 (8.1%)	<.001
Hypertension	747 (66.9%)	272 (69.2%)	257 (64.1%)	218 (67.5%)	0.567
Diabetes mellitus	383 (34.3%)	168 (42.8%)	145 (36.2%)	70 (21.7%)	<.001
Atrial fibrillation	474 (42.4%)	149 (37.9%)	180 (44.9%)	145 (44.9%)	.051
Chronic kidney disease	598 (53.5%)	171 (43.5%)	234 (58.4%)	193 (59.8%)	<.001
Stroke	134 (12.0%)	40 (10.2%)	48 (12.0%)	46 (14.2%)	.097
LVEF (%)	52.1 ± 17.4	47.8 ± 18.1	52.7 ± 17.2	56.5 ± 16.0	<.001
eGFR (ml/min/1.73 m^2^)	46.3 [36.5–68.6]	54.8 [36.5–68.6]	44.4 [31.4–56.8]	42.0 [28.9–56.6]	<.001
BNP (pg/dl)	223 [96–482]	198 [79–479]	233 [102–457]	233 [116–515]	0.996
Prescription at discharge					
RAS inhibitors (ARB or ACE‐I)	688 (61.6%)	268 (68.2%)	235 (58.6%)	138 (57.3%)	.002
β‐blocker	726 (65.0%)	297 (75.6%)	263 (65.6%)	166 (51.4%)	<.001
Diuretic	872 (78.1%)	291 (74.1%)	319 (79.6%)	262 (81.1%)	.02
MRA	180 (16.1%)	74 (18.8%)	63 (15.7%)	43 (13.3%)	.044
Calcium‐channel blocker	234 (21.0%)	79 (20.1%)	86 (21.5%)	69 (21.4%)	0.667
Oral inotropic agent	72 (6.5%)	23 (5.9%)	31 (7.7%)	18 (5.6%)	0.941
Digitalis	50 (4.5%)	19 (4.8%)	20 (5.0%)	11 (3.4%)	0.378
Oral diabetic agent	190 (17.1%)	90 (22.9%)	70 (17.5%)	30 (9.3%)	<.001

*Note*: Categorial data are presented as number (%). Continuous data are presented as mean ± standard deviation (*SD*) or median (Q1 ‐ Q3).

Abbreviations: BMI, body mass index; BNP, brain natriuretic peptide; eGFR: estimated glomerular filtration rate; LVEF, left ventricle ejection fraction; MRA, mineralocorticoid receptor antagonist; RAS, renin‐angiotensin system.

**FIGURE 1:**
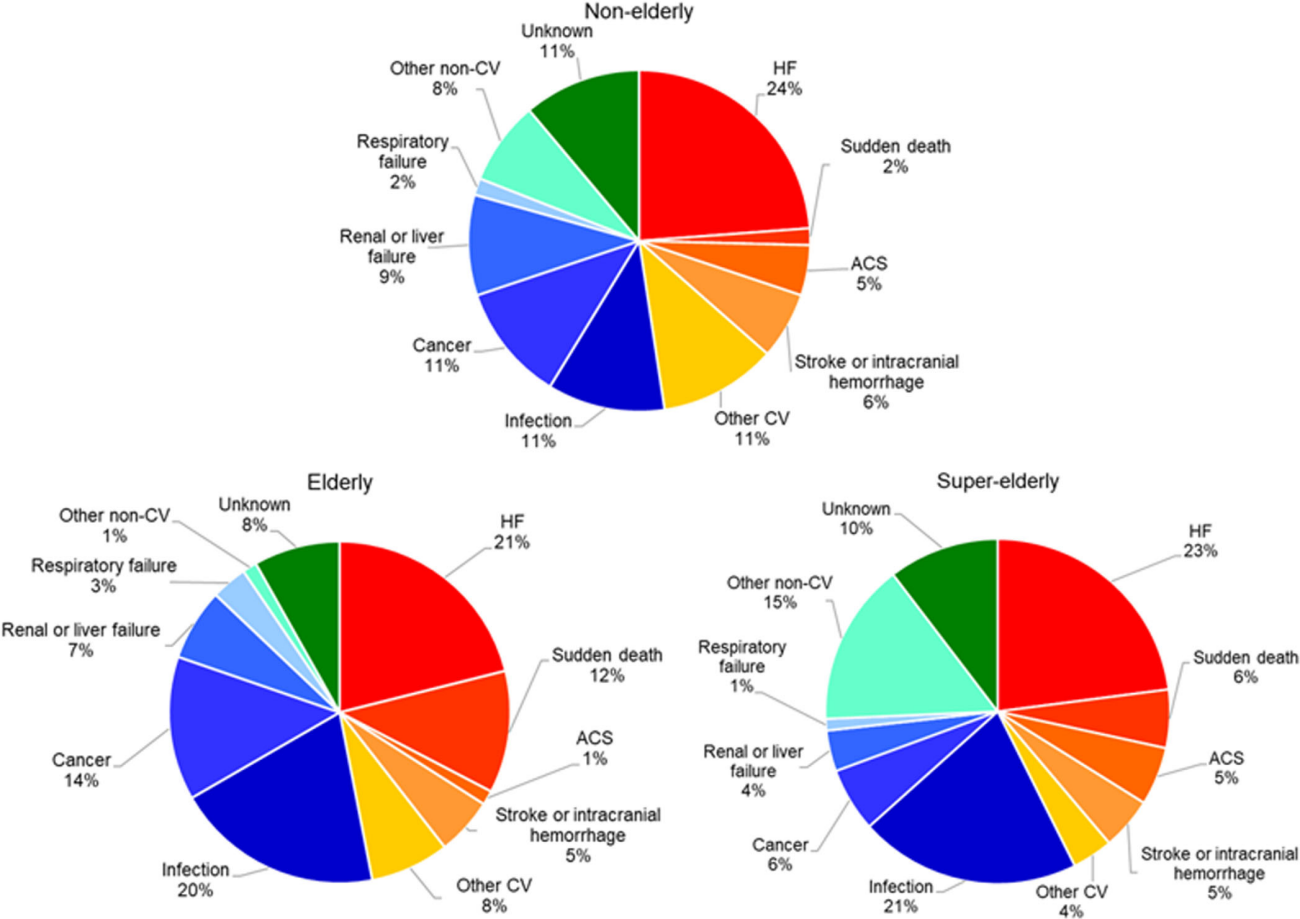
The distribution of the detailed mode of death according to age. Among the three groups, the proportion of HF death was of similar trend; however, the proportion of infection death was higher in elderly and super‐elderly patients. Super‐elderly patients had the lowest proportion of cardiovascular death

Figure 1 shows the distribution of the detailed mode of death according to age. Among the three groups, the proportion of HF death was of similar trend; however, the proportion of infection death was higher in elderly and super‐elderly patients. Super‐elderly patients had the lowest proportion of cardiovascular death (43% vs. 48% in nonelderly and 47% in elderly).

Table 2 shows the incidence numbers and rates of the mode of death in three groups during the follow‐up period. A total of 389 patients (34.8%) had the all‐cause death events, and the highest proportion of the composite endpoint was in super‐elderly, which is 55.4% (179/323). This was followed by 36.7% (147/401) in elderly and 16.0% (63/393) in nonelderly. The annual incidence rate of all‐cause death was 6.04, 16.09, and 29.58 in nonelderly, elderly, and super‐elderly, respectively. In nonelderly, the annual incidence rate of cardiovascular death was higher than that of noncardiovascular death; however, in elderly and super‐elderly, the annual incidence rate of cardiovascular death was lower than that of noncardiovascular death. In all groups, the highest incidence rate of the detailed mode of death was HF death.

**TABLE 2:** Incidence rates of each mode of death during the follow‐up period

	Nonelderly (*N* = 393)			Elderly (*N* = 401)			Super‐elderly (*N* = 323)		
	*N*	Incidence rates (/100 person‐years)	95% CI	*N*	Incidence rates (/100 person‐years)	95% CI	*N*	Incidence rates (/100 person‐years)	95% CI
All‐cause death	63	6.04	5.82–6.27	147	16.09	15.35–16.90	179	29.58	27.61–31.86
Cardiovascular death	30	2.87	2.77–2.99	69	7.55	7.21–7.93	74	12.23	11.41–13.17
HF	15	1.44	1.39–1.49	31	3.39	3.24–3.56	42	6.94	6.48–7.48
Sudden death	1	0.10	0.09–0.10	17	1.86	1.78–1.95	10	1.65	1.54–1.78
ACS	3	0.29	0.28–0.30	2	0.22	0.21–0.23	6	0.99	0.93–1.07
Stroke or intracranial hemorrhage	4	0.38	0.37–0.40	8	0.88	0.84–0.92	9	1.49	1.39–1.60
Other cardiovascular death	7	0.67	0.65–0.70	11	1.20	1.15–1.26	7	1.16	1.08–1.25
Noncardiovascular death	26	2.49	2.40–2.59	78	8.54	8.15–8.97	86	14.21	13.26–15.31
Infection	7	0.67	0.65–0.70	29	3.17	3.03–3.33	38	6.28	5.86–6.76
Cancer	7	0.67	0.65–0.70	20	2.19	2.09–2.30	11	1.82	1.70–1.96
Renal or Liver failure	6	0.57	0.55–0.60	10	1.09	1.04–1.15	7	1.16	1.08–1.25
Respiratory failure	1	0.10	0.09–0.10	5	0.55	0.52–0.57	2	0.33	0.31–0.36
Other noncardiovascular death	5	0.48	0.46–0.50	2	0.22	0.21–0.23	28	4.63	4.32–4.98
Unknown	7	0.67	0.65–0.70	12	1.31	1.25–1.38	19	3.14	2.93–3.38

Abbreviations: ACS, acute coronary syndrome; CI, confidence interval; HF, heart failure.

**FIGURE 2:**
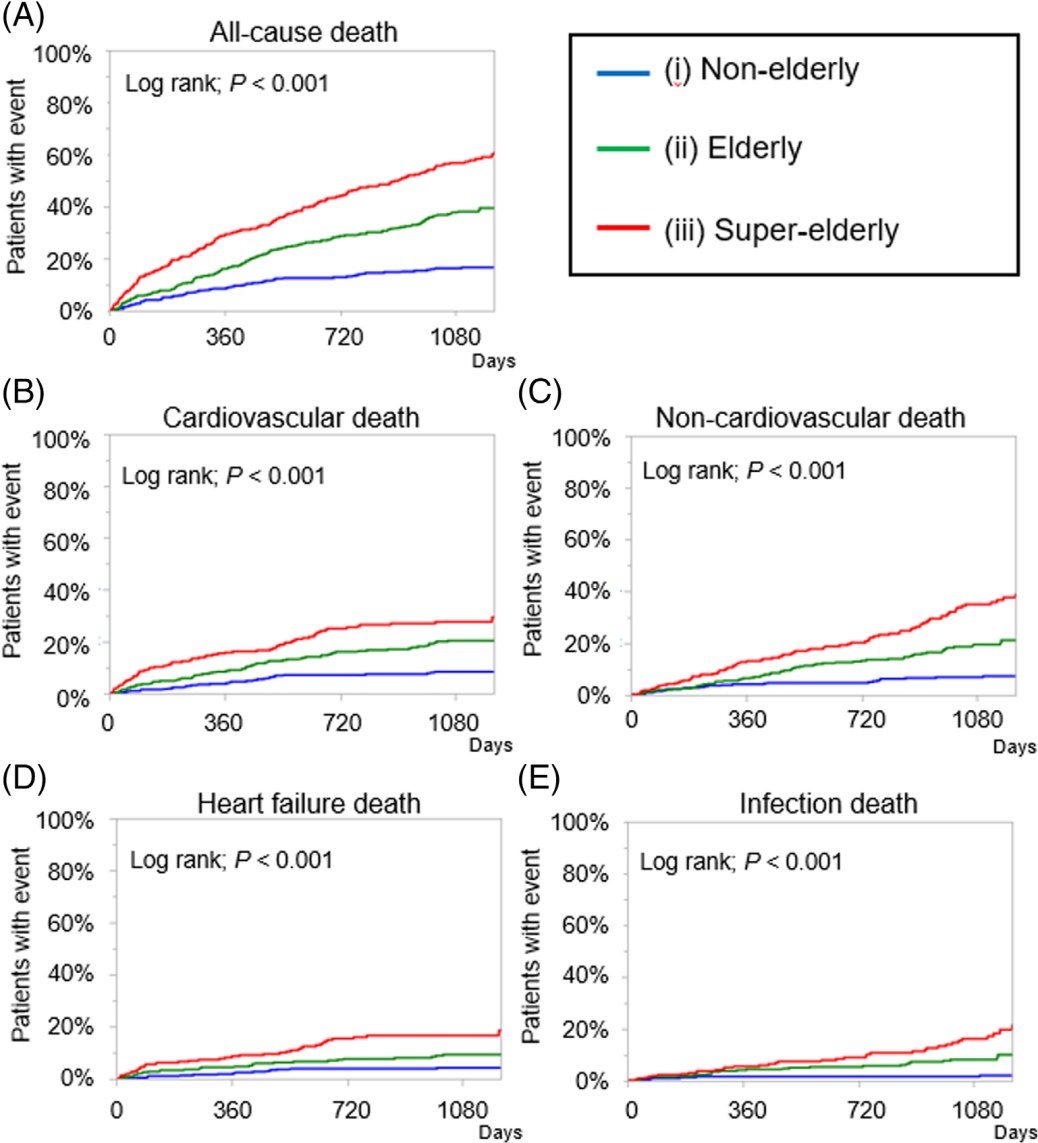
Kaplan–Meier curves for all‐cause death (A), cardiovascular death (B), noncardiovascular death (C), heart failure death (D) and infection death (E) during the 3‐years follow‐up among the following three groups: (i) nonelderly, (ii) elderly, and super‐elderly patients with acute heart failure. In the Kaplan–Meier analysis, a significantly lower rate of the all‐cause death for nonelderly than for the other groups was obtained. As regards the detailed mode of death, significantly decreasing rates of cardiovascular death, noncardiovascular death, HF death, and infection death with decreasing age groups were obtained

During the follow‐up period, the cumulative event rates of all patients were compared among the three groups. Using the Kaplan–Meier method, a significantly lower rate of the all‐cause death for nonelderly than for the other groups was obtained (Figure 2). As regards the detailed mode of death, significantly decreasing rates of cardiovascular death, noncardiovascular death, HF death, and infection death with decreasing age groups were obtained.

In the Cox proportional hazard model (Table 3), the all‐cause death HRs increased significantly in elderly and super‐elderly, when compared with that of nonelderly (adjusted HR, 2.60; 95% CI, 1.93–3.54, p < .001 and adjusted HR, 5.04; 95% CI, 3.72–6.92, p < .001, respectively). Using the Fine–Gray model, the sub‐distribution HRs of cardiovascular death and noncardiovascular death also increased significantly in elderly and super‐elderly patients. Similar tendency was found in the multivariate model adjusted for the other clinical factors including prescriptions at discharge (Table S1). The highest sub‐distribution HR in detailed mode of death was infection death in elderly and super‐elderly patients (adjusted sub‐distribution HR, 4.25; 95% CI, 1.75–10.33, p = .001 and adjusted sub‐distribution HR, 10.10; 95% CI, 3.78–27.03, p < .001, respectively).

**TABLE 3:** Association between age and mode of death compared with nonelderly patients during 3‐years follow‐up

	Nonelderly	Elderly		Super‐elderly	
	Reference	HR (95% CI)	p value	HR (95% CI)	p value
All‐cause death	1.00	2.60 (1.93–3.54)	<.001	5.04 (3.72–6.92)	<.001
Cardiovascular death^a^	1.00	2.19 (1.42–3.38)	<.001	3.55 (2.24–5.65)	<.001
Noncardiovascular death^a^	1.00	2.79 (1.72–4.53)	<.001	5.34 (3.24–8.80)	<.001
HF death^b^	1.00	1.83 (0.98–3.41)	.059	4.87 (2.49–9.50)	<.001
Other cardiovascular death (without HF death^b^)	1.00	2.44 (1.33–4.48)	.004	2.26 (1.21–4.20)	.010
Infection death^b^	1.00	4.25 (1.75–10.33)	.001	10.10 (3.78–27.03)	<.001
Other noncardiovascular death (without infection death^b^)	1.00	2.08 (1.15–3.77)	.016	3.22 (1.83–5.67)	<.001

*Note*: HR of adjusted model was adjusted for sex and co‐morbidities (yes/no); history of heart failure, coronary artery disease, valvular disease, cardiomyopathy, hypertension, diabetes mellitus, atrial fibrillation, chronic kidney disease, and stroke.

^a^ Sub‐distribution hazard ratio for cardiovascular death and noncardiovascular death was estimated by Fine‐Gray model between cardiovascular and noncardiovascular death.

^b^ Sub‐distribution hazard ratio for HF death, other cardiovascular death without HF death, infection death and other noncardiovascular death without infection death was estimated by Fine‐Gray model among four groups. HR: hazard ratio, CI: confidence interval, HF heart failure.

## 4 DISCUSSION

In this prospective registry of AHF patients in Japan, the elderly and super‐elderly patients had a significantly greater risk of reaching all‐cause death after discharge among AHF patients. Additionally, this study revealed that noncardiovascular death, especially infection death, was a more common risk in elderly and super‐elderly AHF patients than cardiovascular death. These differences were independently maintained despite adjustments for differences in comorbidities and prescriptions. To our knowledge, this is the first prospective report to examine the association between super‐elderly and prognosis and include information that is helpful for preventative approaches and management for patients following AHF.

This study found that the risk of all‐cause death in AHF patients with elderly was 2.5‐fold higher, and that with super‐elderly was more than fivefold higher than that with nonelderly. Interestingly, a gradual increased risk was found more in noncardiovascular death than in cardiovascular death. Moreover, the major noncardiovascular death was caused by infection death. Especially, the risk of infection death in AHF patients with super‐elderly was tenfold higher than that with nonelderly. These results were obtained from previous studies.^10^, ^11^ The NARA‐HF study, a single‐center retrospective study, reported that no significant differences were observed in cardiovascular death between patients aged <75 years and those aged ≥75 years with AHF, but a risk of noncardiovascular death, mostly caused by infection, in those aged ≥75 years was significantly higher than in those aged <75 years.^11^ This previous study showed that adjusted HR for noncardiovascular death was 3.25 and that for infection death was 7.09 in patients aged ≥75 years in comparison with those >75 years old. Our study revealed that the HR for noncardiovascular death was 2.79 and 5.34 in elderly and super‐elderly patients with AHF, respectively. Furthermore, the HR for infection death was 4.25 and 10.1 in elderly and super‐elderly patients with AHF, respectively. The risk for infection death in super‐elderly was 2.3‐fold higher than that in elderly patients. These findings suggest that patients with AHF should have managements to prevent infections, to improve the outcomes after discharge. In this study population, 73.0% (*N* = 54/73) of the infection death was caused by pneumonia. A previous study showed that influenza vaccination was associated with lower all‐cause death even after the adjustment for the propensity score matching in patients with HF.^18^ Another large nationwide cohort study in Denmark showed that influenza vaccination was associated with a reduced risk of all‐cause and cardiovascular deaths in patients with HF; moreover, frequent vaccination and vaccination earlier in the year were associated with reduced risk of death compared with intermittent and late vaccination.^19^ Prior vaccination against pneumococcus is obviously associated with improved survival and decreased chance of respiratory failure in elderly people.^20^ Furthermore, both influenza and pneumococcus vaccination together demonstrated added benefits in elderly patients.^21^ In 2020, COVID‐19 pandemic has occurred, and elderly, HF, and cardiovascular disease were the severe risk factors of mortality.^22^ Unfortunately, this registry has no detailed data on pneumonia; however, elderly and super‐elderly patients with HF should take some vaccinations by winter season.

In our study population, 35.9% and 28.9% belonged to the elderly and super‐elderly patients with AHF, respectively. A recent prospective study in Spain conducted a similar study that evaluated an association between 1‐year clinical outcomes and super‐elderly patients with AHF.^12^ They showed that 1‐year all‐cause death rates in nonelderly, elderly, and super‐elderly groups were 14.0%, 24.4%, and 42.3%, respectively, and 1‐year HF death rates in nonelderly, elderly, and super‐elderly patients with AHF were 5.1%, 11.8%, and 19.6%, respectively. In Western countries, an annual death rate in hospitalized patients with AHF reached approximately 20%.^23^, ^24^ Major HF cohort studies in Japan reported approximately 7%–20% annual mortality in patients with HF.^10^, ^25^, ^26^ This study showed that 1‐year all‐cause death rates in nonelderly, elderly, and super‐elderly groups were 6.4%, 16.1%, and 29.6%, respectively, and 1‐year HF death rates in nonelderly, elderly, and super‐elderly patients with AHF were 1.4%, 3.4%, and 6.9%, respectively. This study included a higher proportion of elderly and super‐elderly patients than previous Japanese cohort studies. Although there were several differences in medical systems, management, and characteristics between Japan and Western countries, Japanese patients with AHF might have the potential to better prognosis than those in Western countries. However, mortality in Japanese patients with AHF was still high, one‐third of the super‐elderly patients died in 1 year, which reinforces the importance of advance care planning (ACP) and palliative care in patients with HF. Although tools for ACP and palliative care management are more pervasive in Western countries than in Japan, some Japanese tools for ACP and palliative care management recently exist.^27^, ^28^

The Spanish cohort also showed that adjusted HRs of HF death was 2.02 in elderly patients and 3.32 in super‐elderly patients, compared with those of <65‐year‐old patients.^12^ The HR of HF death in our study was similar to their results (adjusted HR: 2.19 and 3.55 in elderly and super‐elderly, respectively). HF death rate in patients with AHF was also still high in elderly and super‐elderly patients. Several studies showed that the positive effects of physical rehabilitation in patients with chronic HF were confirmed.^29^, ^30^ A nurse‐led, multidisciplinary home‐based intervention after discharge of AHF contributed to the decreasing risk of mortality.^31^ Malnutrition was a high risk of mortality in patients with HF,^32^ and nutrition therapy is also important to prevent adverse outcomes in patients with HF. Several tools should be applied more to elderly and super‐elderly patients with AHF according to each individual condition.

This registry has several limitations. First, this study was a post‐hoc analysis using a prospective cohort study with inherent associated limitations. Second, detailed data of post‐discharge therapy and intervention, such as the implementation of cardiac rehabilitation, nutrition scores, and home‐based care, were limited. In patients with HF, cardiac rehabilitation, including nutrition counseling and nursing care, involves the heart team staff and is one of the most important treatments to improve outcomes in patients with AHF. Third, this registry enrolled consecutive patients with HF in each hospital, but few patients might be able to give or get informed consent. However, this cohort study could be achieved to collect the targeted number of patients before the targeted period. Finally, the mode of death was difficult to classify on some patients, especially in elderly and super‐elderly patients, even if complete information was obtained. Although there were several limitations, we believe that the results are useful, and the information are helpful in the clinical practice in elderly and super‐elderly patients with HF.

## 5 CONCLUSION

In this population, the risk of all‐cause death was found to have a gradually increase in elderly and super‐elderly patients, when compared with nonelderly patients. Elderly patients, especially super‐elderly patients, with AHF were at a higher risk for noncardiovascular death, especially infection death. They should be managed to prevent not only following cardiovascular events but also infections using medical resources and their supporters.

## CONFLICT OF INTEREST

The authors declare that there is no conflict of interest.

## Supporting information


**Table S1** Association between age and mode of death compared to nonelderly patients adjusted for the prescriptions.

## Data Availability

The deidentified participant data will be shared on a request basis. Please directly contact the corresponding author to request data sharing. The data contained the baseline data follow‐up data of patients and the study protocol in Japanese. The data is available immediately.
