# Supplementary material for: Mode of death in elderly and super‐elderly patients with acute heart failure: Insights from Japanese heart failure registry
Source: Clin Cardiol. 2021 May 8;44(6):848–56. doi: 10.1002/clc.23619 (PMC8207972; doi:10.1002/clc.23619)
Supplement: Supplementary file 1 — Table S1 Association between age and mode of death compared to nonelderly patients adjusted for the prescriptions. [file CLC-44-848-s001.docx]

| **Table S1. Association between age and mode of death compared to non-elderly patients adjusted for the prescriptions** | | | | | | | |
| --- | --- | --- | --- | --- | --- | --- | --- |
|  |  |  |  |  |  |  |  |
|  | Non-elderly |  | Elderly | |  | Super-elderly | |
|  | *Reference* |  | *HR (95% CI)* | *P value* |  | *HR (95% CI)* | *P value* |
| All-cause death | 1.00 |  | 2.51 (1.87 - 3.42) | <0.001 |  | 4.68 (3.47 - 6.39) | <0.001 |
| Cardiovascular death* | 1.00 |  | 2.20 (1.43 - 3.37) | <0.001 |  | 3.56 (2.31 - 5.50) | <0.001 |
| Non-cardiovascular death* | 1.00 |  | 2.50 (1.54 - 4.05) | <0.001 |  | 4.17 (2.59 - 6.70) | <0.001 |
| HF death † | 1.00 |  | 1.62 (0.86 - 3.03) | 0.14 |  | 3.63 (1.94 - 6.80) | <0.001 |
| Other cardiovascular death   without HF death † | 1.00 |  | 2.73 (1.52 - 4.93) | <0.001 |  | 3.06 (1.68 - 5.57) | <0.001 |
| Infection death † | 1.00 |  | 4.11 (1.84 - 9.14) | <0.001 |  | 7.81 (3.61 - 16.92) | <0.001 |
| Other non-cardiovascular death   without infection death † | 1.00 |  | 1.84 (0.99 - 3.45) | 0.055 |  | 2.68 (1.48 - 4.86) | 0.001 |
|  |  |  |  |  |  |  |  |
| HR of adjusted model was adjusted for sex, eGFR, BNP and prescriptions (yes/no); RAS inhibitors, β-blocker, diuretic, MRA, calcium-channel blocker, oral inotropic agent, digitalis, and oral diabetic agent. *Sub-distribution hazard ratio for cardiovascular death and non-cardiovascular death was estimated by Fine-Gray model between cardiovascular and non-cardiovascular death. †Sub-distribution hazard ratio for HF death, other cardiovascular death without HF death, infection death and other non-cardiovascular death without infection death was estimated by Fine-Gray model among 4 groups. HR: hazard ratio, CI: confidence interval, HF heart failure. | | | | | | | |
